# Comparative Performance Analysis of an Open Source Motion‐Compensated Cardiac Diffusion Sequence Across Different MRI Systems and Gradient Strengths—A Pilot Study

**DOI:** 10.1002/mrm.70527

**Published:** 2026-07-19

**Authors:** Eric Arbes, Ariel Hannum, Johannes Fischer, Qingping Chen, Sebastian Littin, Alexander Maier, Michael Bock, Simon Reiss

**Affiliations:** ^1^ Division of Medical Physics, Department of Diagnostic and Interventional Radiology Medical Center – University of Freiburg, Faculty of Medicine – University of Freiburg Freiburg Germany; ^2^ Department of Radiology Stanford University Stanford California USA; ^3^ Department of Cardiology and Angiology University Heart Center Freiburg‐Bad Krozingen, Faculty of Medicine ‐ University of Freiburg Freiburg Germany

**Keywords:** cardiac diffusion imaging, diffusion tensor imaging, open source, vendor‐independent pulse sequence programming

## Abstract

**Purpose:**

To validate the performance of an open‐source cardiac diffusion sequence framework and to investigate hardware‐based performance differences.

**Methods:**

Three second order motion compensated sequence files were compiled using Pulseq and applied across different MRI systems to image five volunteers. DTI and SNR analysis was performed to investigate protocol efficacy.

**Results:**

The sequences performed well at the task of imaging short axis views of the myocardium and producing data suitable for diffusion tensor imaging analysis. Significant differences in sequence performance across different systems and within the same system were revealed. Relative SNR utilizing the strongest available gradient system yielded up to 80% more SNR than the weakest tested system. Relative SNR obtained from the same sequence at different scanners differed by up to 42%, suggesting hardware impacts that warrant further investigation.

**Conclusion:**

The presented sequence framework allows for an easy starting point into cardiac diffusion tensor imaging and opens the door to further analysis of hardware impacts on performance.

## 1 Introduction

Cardiac diffusion magnetic resonance imaging (MRI) allows to probe myocardial microstructure, enabling the detection of abnormalities such as myocardial infarctions [1, 2], cardiomyopathy and congenital heart disease [3]. Diffusion weighted imaging (DWI) and diffusion tensor imaging (DTI) are well established in brain imaging, but in the heart, bulk motion from the heart beating and respiration leads to motion‐induced signal loss during the strong diffusion‐sensitizing gradients [4]. To minimize signal dephasing due to bulk motion, cardiac diffusion sequences typically utilize spin‐echo (SE) acquisition modules with higher‐order gradient moment nulling [5] up to the second order (M1 + M2), with some studies suggesting that even a third order moment nulling may provide additional robustness [6]. Gradient moment nulling is realized through additional diffusion gradient lobes which require more time for diffusion encoding [7]. This time penalty is usually on the order of 10s of milliseconds per moment order [7]—the time decreases with increasing gradient amplitude and slew rate of the utilized gradient system. Strong gradient systems allow for shorter gradient waveforms thus reducing the echo time (TE) [8]; as the transversal relaxation time (T2) of the human myocardium is on the order of 50–60 ms at 3T [9], a reduction in TE is beneficial as it will minimize additional signal loss due to T2 relaxation. Recently, clinical whole‐body MRI systems have become available with gradient strengths up to 200 mT/m that can facilitate in vivo diffusion imaging of the myocardium [10]. Recent studies show that these systems enable signal‐to‐noise ratio SNR gains of up to 40% for second order motion‐compensated cardiac DTI sequences [11].

From the individual diffusion‐weighted myocardial images parameters such as the mean diffusivity (MD), the fractional anisotropy (FA), and principal eigenvector orientations (*e*_1_) are calculated; however, these values depend on the exact sequence implementation and the properties of the MRI system used. For example, fat saturation protocols are usually vendor‐specific and are highly impactful in cDTI due to the strong chemical shifts of fat signal in phase encoding direction inherent to single shot echo planar imaging (EPI) [12] and the relative weakening of water signal compared to fat inherent to diffusion weighted imaging [13]. Exact pulse shapes and phases of excitation and refocusing pulses also tend to differ, which has an impact on slice profiles, potential spin‐history effects, level of heating in tissue and refocusing efficiency [14]. Thus, it is difficult to compare data sets from different MRI systems quantitatively without precise knowledge of the details of the sequence, and the longitudinal evaluation of the changes of myocardial substructures and abnormalities under therapy might become challenging.

In this work, the open source vendor‐agnostic sequence development platform Pulseq [15] is used to develop a second order motion compensated cardiac diffusion SE sequence that can acquire DTI data with identical sequence timing at different MRI systems [16]. Utilizing this system independent approach which removes the common confounds of specific sequence implementations discussed prior, this work seeks to isolate potential hardware specific impacts on DTI quantities such as differing gradient impulse responses, B0 and B1 shims, and frequency calibrations [17, 18, 19]. This approach also extends to subsequent image reconstruction, where raw data is used to directly reconstruct with open‐source code instead of taking precomputed DWIs from scanner specific reconstruction pipelines. The sequence is tested in volunteers at three different clinical MR systems to perform a quantitative analysis of the effect of system performance on quantitative cardiac DTI results.

## 2 Methods

### 2.1 Sequence Design

Three different versions of a second order motion compensated cardiac spin‐echo MRI sequence were designed using the open source Pulseq environment. Sequences were implemented with three different maximum gradient amplitudes of *G*_max_ = 200 mT/m (Sequence 1), 80 mT/m (Sequence 2) and 45 mT/m (Sequence 3) and a common maximum slew rate of *s*_max_ = 200 mT/m/ms. To minimize TE while ensuring the compensation conditions $\int G\left(t\right)t^{n}\mathrm{dt}=0,n=0,1,2$, gradient rise times and amplitudes were calculated based on provided hardware limits for any given desired trapezoidal gradient lobe. Due to peripheral nerve stimulation (PNS) limitations, the diffusion gradient lobes could not always exploit the gradient hardware limits. In cases where PNS limits were reached, the slew rate of the diffusion gradients was reduced while keeping the amplitudes consistent.

The EPI readouts were designed with maximum gradient settings, a GRAPPA [20] acceleration factor of 3, a partial Fourier factor of 6/8 and ramp sampling. Nine diffusion sensitizing directions with a calculated [21] weighting of *b* = 350 s/mm^2^ were implemented according to the golden means principle [22]. Acquisitions of all nine directions were performed across three breath holds with three directions each including a reference line scan without phase encoding blips, fully sampled autocalibration lines for GRAPPA reconstruction of the undersampled *k*‐space data sets and a reference diffusion acquisition with *b*_ref_ = 50 s/mm^2^ weighted along the slice selection direction. The reference lines were used for linear and constant EPI phase correction [23]. As shown in the sequence schematic (Figure 1), additional fat suppression was realized with short‐TI inversion recovery (STIR) pulse placed 220 ms before the 90° RF excitation pulse, followed by bipolar second order motion compensated diffusion sensitizing gradients placed around the 180° refocusing pulse and an echo planar imaging (EPI) readout. To minimize artifacts from surrounding adipose tissue, slice selection gradients were applied in orthogonal directions relative to each other to only excite the central 50% of the slice volume [24]. The size of the sampled FOV was not changed as no further reduction of TE could be obtained by shortening the echo train. This was due to the diffusion gradients being constrained by PNS limits. All individual images were cycled across three different slices on each heart beat with a voxel size of 2.3 × 2.3 × 8 mm^3^ and a gap between slices of 3 mm. A FOV of 300 × 300 mm, a readout bandwidth of 1515 Hz/px and an echo spacing of 0.7 ms were used.

**FIGURE 1:**
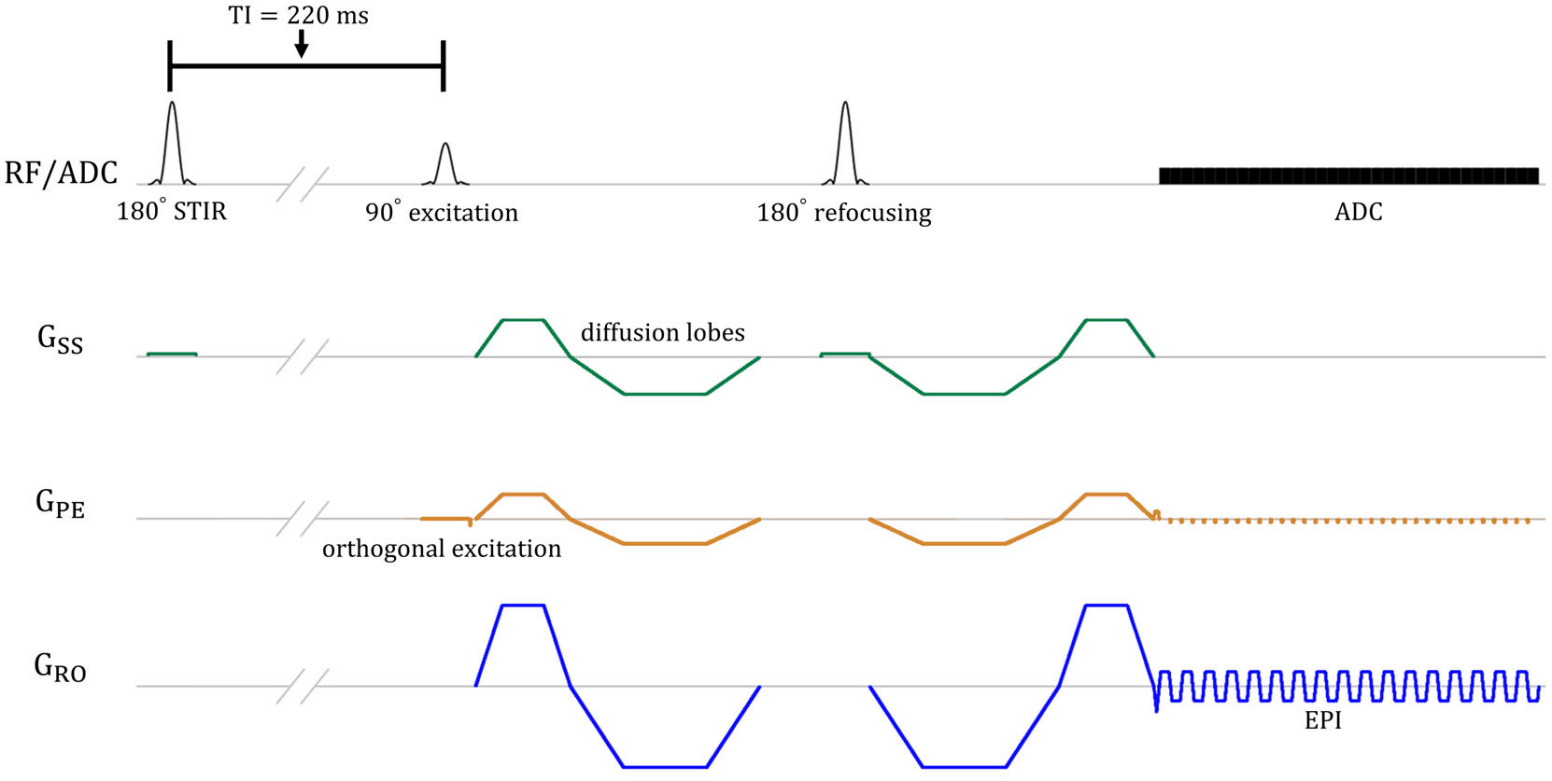
Sequence diagram. A STIR pulse precedes the excitation pulse to remove unwanted signal. The slice selection gradient of the excitation pulse is placed in the phase encoding direction to only excite the inner half of the FOV. Diffusion lobes are bipolar and follow a motion compensated design. The EPI readout is kept short through the use of GRAPPA and partial Fourier imaging.

### 2.2 Phantom Study

In order to obtain a baseline comparison for DTI performance, a diffusion phantom produced in‐house was imaged at three different 3T MRI systems corresponding to Sequences 1–3 in terms of their maximum gradient amplitudes (Cima.X (System 1), Prisma.Fit (System 2), Vida (System 3), Siemens Healthineers, Erlangen Germany) with all possible sequence combinations. Details of the systems and respective sequences are listed in Table 1. The sequences used of for phantom imaging were identical to the ones used in vivo. The maximum slew rates were used for the imaging gradients, that is, the EPI readout, but they were not feasible for the diffusion gradient lobes due to PNS constraints in all cases. The achieved TEs were 50 ms for sequence 1, 67 ms for sequence 2 and 79 ms for sequence 3 due to tighter PNS constraints. Figure S2 shows a plot of achievable TEs depending on the target b values at the three different systems. The diffusion phantom consisted of tubes suspended in liquid to avoid susceptibility‐induced artifacts (Figure 2A). Different diffusivities within the tubes were achieved through the use of aqueous polyvinylpyrrolidone (PVP) [25], with at least two tubes being prepared per targeted value for the diffusivity, ranging from 0.54 to 1.99 $\mu m^{2}/\mathrm{ms}$. A high precision balance was used to replicate the literature PVP concentrations associated with the different diffusivities at different temperatures. The phantom was stored inside an MR cabin for multiple days before the measurements for temperature adaptation. The target temperature of the MR cabins of the three systems is set to 19°C, however, a temperature uncertainty of ±1°C was assumed for the calculation of the errors of the MD values of the phantom. Five averages of nine diffusion directions and one reference image were acquired for DTI analysis. A synthetic ECG signal of 60 bpm was used as a trigger signal. A product SE‐EPI sequence with a *b* value of 1000 s/mm^2^ and monopolar diffusion gradients using the same FOV and resolution was acquired as a reference.

**TABLE 1:** Used sequences and their properties.

Sequence	Max. grad (mT/m)	Max. Diff. slew rate (mT/m/ms)	Max. slew rate (mT/m/ms)	TE (ms)	Used at system 1	Used at system 2	Used at system 3
1	200	120	200	50	Yes	No	No
2	80	50	200	67	Yes	Yes	No
3	45	45	200	79	Yes	Yes	Yes

**FIGURE 2:**
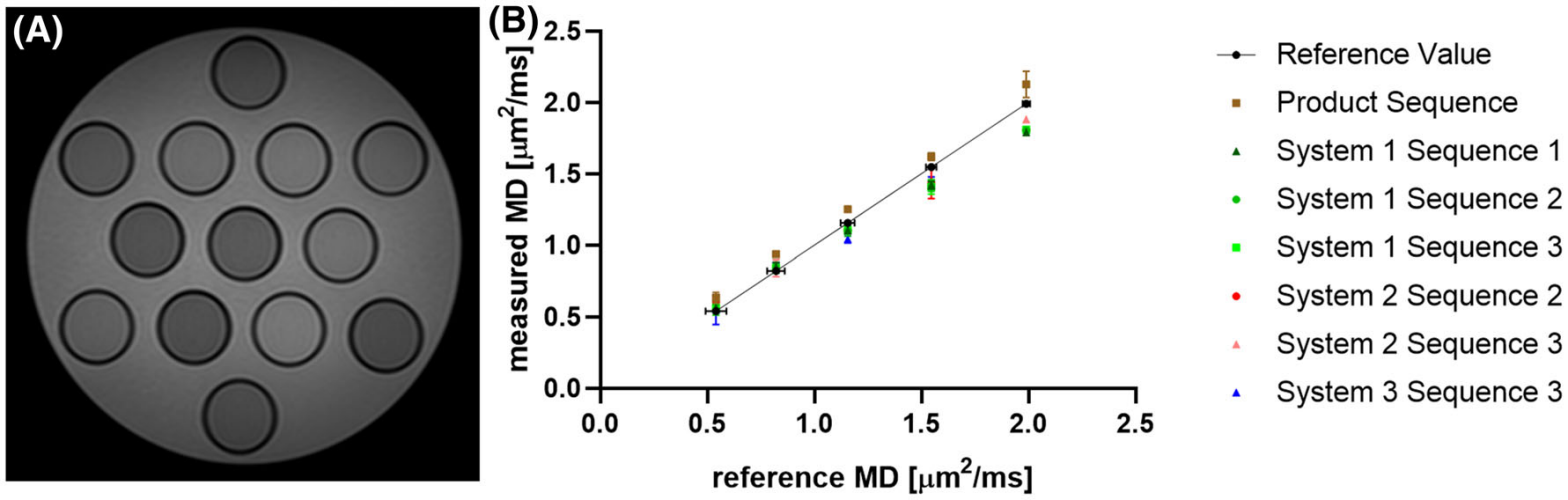
(A) Localizer image of the diffusion phantom. The same level of diffusivity was present in either pairs of two or three tubes. (B) Measured MD versus reference MD for all sequence and scanner combinations used in this work as well as a product sequence. Values were obtained from ROI means for each tube.

### 2.3 Comparative Volunteer Measurements

Five volunteers (3/2 male/female, age: 27 ± 4 years) were imaged at the same three different 3 Tesla MRI systems as the phantom study after written informed consent was obtained. Volunteer experiments were approved by the institutional review board of the University Medical Center Freiburg (No. 160/2000). The volunteers were presumed healthy due to no current or past relevant reported health issues, with no additional health screening being performed. The volunteers reported moderate levels of physical activity, and no ECG abnormalities were observed. The average time between scans for an individual subject was 9.3 ± 9.7 days (minimum/maximum: 2/33 days), with the order of systems at which the volunteers were measured being randomized. The volunteers had an average BMI of (20.74 ± 2.97) kg/m^2^ and the weight of the volunteers was observed to be stable within this timeframe. None of the volunteers reported use of regular medication, arterial hypertension, past or present smoking habits, diabetes, history of cardiovascular disease, or hyperlipidemia. Two of the volunteers reported family history of cardiovascular events. An 18‐channel body array receive coil was used for signal reception together with a 32‐channel spine coil. All images were acquired in short axis orientation of the myocardium, with phase encoding in right to left direction. The time delay after the R‐wave for the acquisition of the short axis reference images was matched to the timing of the cDTI sequences for reference. Timing of the cDTI acquisition was not adjusted within one imaging session since the duration of the systole was assumed to be robust against changes in heart rate [26]. The acquisition was split across three breath holds of 18 heart beats each, containing three diffusion sensitizing directions per slice (9 heart beats total), each with a unique reference scan per slice to adapt for changes of the position of the heart between breath holds (3 heart beats in total), as well as the three line navigators and autocalibration line scans per slice (3 heart beats each). The sets of breath holds were repeated 5 times for averaging. The acquisition was ECG‐triggered with a delay of 5 ms after the R‐wave, leading to a total delay of 225 ms with the STIR pulse. The total scan time was 10 min per acquisition, with 4.5 min taken up by breath holds, assuming a standard heart rate of 60 bpm and the rest of the time going to recovery between breath holds. Heart rates of all volunteers were between 45 and 70 bpm during imaging.

A retest utilizing the same sequence protocol for sequence 1 and system 1 was performed in all volunteers to test reproducibility. Additionally, a measurement was performed in the same volunteers for sequence 1 and system 1 with changes made to the sequence protocol in order to investigate their impact. These measurements were acquired in free‐breathing, with the amount of diffusion directions increased to 20 and the number of averages increased to 10. An echo time of 55 ms was used and the total scan time was 11 min. The data was retrospectively gated to only include acquisitions during exhalation, yielding 3 useable averages per diffusion direction on average. The *b* value was increased to 400 s/mm^2^ and *b*_ref_ was changed to have equal components in the *x*, *y* and *z* axes. In addition, the “flip‐back” method as validated by Luo et al. [27] was implemented to potentially improve image quality for multislice imaging with reduced FOV and cine acquisitions were used to better match the delay time of the trigger to the individual end systolic phases.

### 2.4 Reconstruction and Data Analysis

Diffusion‐weighted images were reconstructed from the raw data and post‐processed using an in‐house code implemented in MATLAB (R2024a, The MathWorks Inc., Natick, USA) [28]. Ramp‐sampled data points were resampled through linear interpolation and constant and linear phase corrections [23] were derived from the reference data. GRAPPA reconstruction was done with the grappa‐tools toolbox [29]. DTI post‐processing was performed with the CarDpy toolbox [30, 31], that called DIPY functions. Post‐processing steps included Gibb's Ringing Correction [32, 33] followed by affine registration, averaging, interpolation from a resolution of 2.3 × 2.3 × 8.0 mm to 1.15 × 1.15 × 8 mm using *k*‐space zero‐filling [34] and then Local PCA denoising [35, 36]. A non‐linear least squares diffusion tensor model was fitted and then pixel‐wise MD, FA [37], and *e*_1_ orientation maps were obtained. The normalized e_1_ vector was projected onto the circumferential‐longitudinal plane in the LV mask to generate helix angle maps. The pipeline was verified using publicly available reference data [38]. Regional mean values of cDTI metrics were obtained by manually drawing an ROI of the whole left ventricular (LV) myocardium in each image in accordance with consensus procedures [10]. In slices where artifacts were present, the relevant areas were excluded from the ROI. A two‐tailed paired *t*‐test was used to test significance (*p* < 0.05) between the different MD and FA data sets. Trace images were used for qualitative evaluation of acquisitions and obtained by calculating the geometric mean of the individual diffusion‐weighted images [39].

To quantify data quality, a bootstrapping [40, 41] method was applied. Since every slice was acquired with 9 different diffusion weighting directions and 5 averages, unique data set permutations could be derived by randomly assigning one of the averages to each diffusion direction. In this way, 400 individual MD, FA and E1 maps were derived from each slice data set [42]. MD and FA uncertainties were quantified by calculating the voxelwise two‐sided 95% confidence intervals of the bootstrapped maps. The derived values are referred to as dMD and dFA and respectively. Uncertainty of e1 (dE1) was computed by calculating the width of the one‐sided 95% interval from the bootstrapped diffusion tensor. Histograms of each voxel in the manually segmented LV myocardium were used to determine the maximum likelihood uncertainty value of each slice as well as the median. Their respective bin sizes were 0.05 $\mu m^{2}/\mathrm{ms}$, 0.05 a.u. and 5°. These maximum values and medians were averaged across the different slices and volunteers for each sequence and scanner combination. Values that exceeded three times the median across the different scanner and sequence combinations were excluded to account for potential artifact regions that could not be segmented out for each bootstrapping iteration due to the size of the dataset.

Temporal SNR (tSNR) and relative SNR (rSNR) were calculated from the magnitude images of the *b* = 350 s/mm^2^ data since the non‐uniformity of noise distributions in parallel imaging renders standard region of interest (ROI) based methods inaccurate [43]. tSNR across the averages was calculated from the individual acquisitions by dividing the pixelwise mean signal $\bar{S}$ by the standard deviation σ across the series of acquisitions: $\text{tSNR}=\bar{S}/\sigma$ [44, 45]. tSNR was only considered from manually drawn ROIs of the left ventricular wall. A two tailed paired *t*‐test was used to test significance between the different acquisition setups. As only averages of the same acquisition are examined, tSNR is a measure of signal stability over time.

rSNR between two images with $S_{1},\sigma_{1}$ and $S_{2},\sigma_{2}$ was calculated via $\text{rSNR}=\frac{S_{1}/\left(\frac{g_{1,S}}{g_{1,\sigma }}\sigma_{1}\right)}{S_{2}/\left(\frac{g_{2,S}}{g_{2,\sigma }}\sigma_{2}\right)}$ [45], where σ is a noise ROI, and *g* is the geometry factor of the signal or noise areas. When using identical coil geometry and consistent ROIs, the *g* factors of the different acquisitions can be assumed to be equal, simplifying the expression to $\text{rSNR}=\frac{S_{1}\sigma_{2}}{S_{2}\sigma_{1}}$ [45]. The same noise region in the top right corner of the FOV was used for all images. Since only images with the same *g* factors are compared, rSNR allows for the use of ROI based comparisons assuming that the ROI positions are equal in both matrices. rSNR was determined pixelwise and a final local average rSNR number was subsequently derived from a manually drawn ROI of pixels within the left ventricle for each given slice. rSNR was compared between the 3 system configurations by averaging images of each diffusion direction. Due to the non‐normal distribution properties of ratios, significance was tested using a one‐tailed Wilcoxon signed rank test for data acquired at the same machine and a two‐tailed test was used for data of the same sequence across the different scanners.

## 3 Results

### 3.1 Sequence Performance

Example images from one volunteer are shown in Figure 3. The first averages of *b*_ref_ and the first six diffusion weighted images are shown for all sequence and system combinations. Subcutaneous fat signal is mostly suppressed, with residual signal being particularly visible in the outer chest wall. Flow signal is visible in the ventricles in the *b*_ref_ images, which is more prominent in the basal slices. The myocardial shape appears minimally distorted in all acquisitions due to the reduction in acquisition lines through partial Fourier sampling and parallel imaging.

**FIGURE 3:**
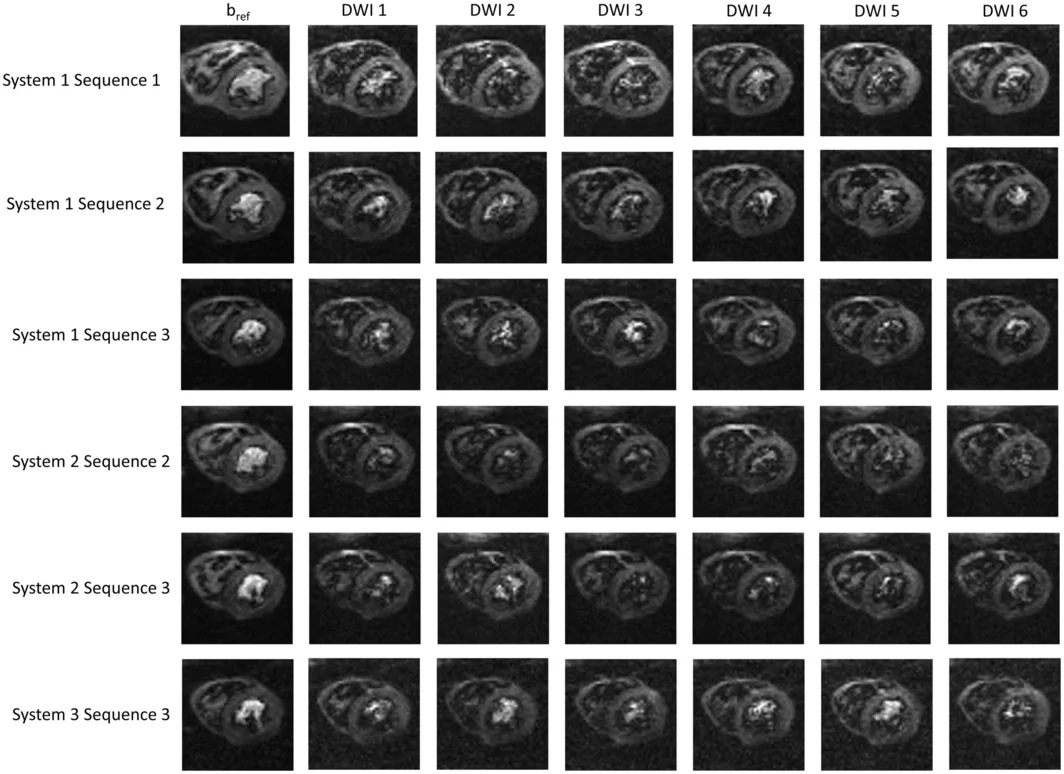
First averages of the reference measurement and first six diffusion weighted images for each system and sequence combination.

### 3.2 DTI Results

MD values obtained in the diffusion phantom are shown in Figure 2B. The product SE‐EPI sequence shows systematically higher MD values compared to the designed reference values with an average deviation of (9.55 ± 5.20) %. The maximum difference between MD values obtained from all possible sequence and scanner combinations is 0.09 $\mu m^{2}/\mathrm{ms}$ (4.7%) for a reference MD value of 1.99 $\mu m^{2}/\mathrm{ms}$, which was found between sequence 3 at system 2 and sequence 1. MD values obtained with the proposed sequence show a slight underestimation of MD, which becomes more pronounced at higher MD reference values. Comparing the averages of all sequence and scanner combinations to the references for the MD values that are close to the values expected in the myocardium (1.16 and 1.55 $\mu m^{2}/\mathrm{ms}$) yields a deviation of 4.9% and 7.6%, respectively. Figure S3 shows the data for each system separately.

Figure 4 shows MD and FA maps in one slice for the different sequences and systems in one volunteer. Figure 5 shows the averages of the MD and FA for all volunteers, slices, sequences and systems. Reference values for mid ventricular short axis slices in mid systole of healthy subjects were taken from literature and marked in the figures. The measured MD averages generally agree with literature values within the error range of (145 ± 0.23) $\mu m^{2}/\mathrm{ms}$ [10]. The most significant intra‐sequence difference was observed between sequence 1 at system 1: (1.49 ± 0.08) $\mu m^{2}/\mathrm{ms}$, and sequence 3 at system 1: (1.41 ± 0.24) $\mu m^{2}/\mathrm{ms}$.

**FIGURE 4:**
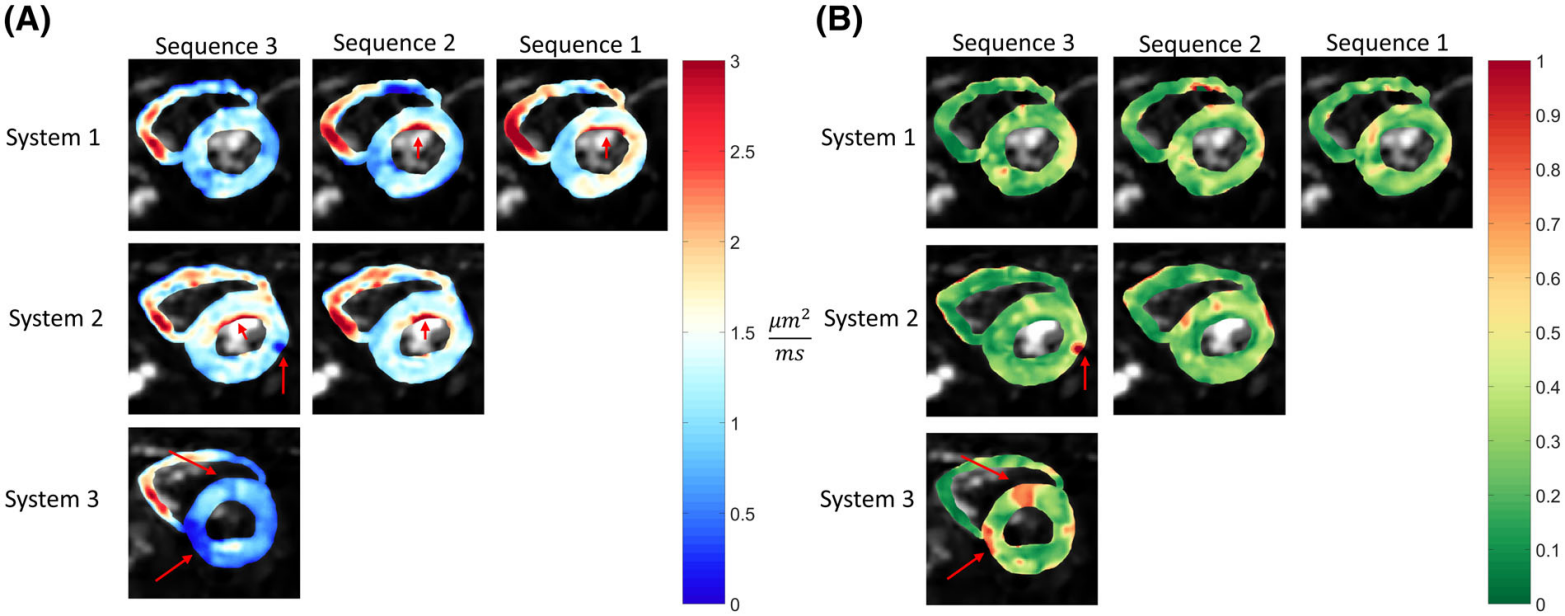
(A) Example MD maps of one volunteer. The right ventricle is shown for demonstration purposes but was not included in further quantitative analysis. (B) Example FA maps of one volunteer. The right ventricle is shown for demonstration purposes but was not included in further quantitative analysis. Observed artifacts are marked with red arrows. Residual fat artifacts often presented themselves as elevated levels of MD or FA in the epicardial areas. Endocardial elevation of MD presumably was caused by blood pool signal contamination.

**FIGURE 5:**
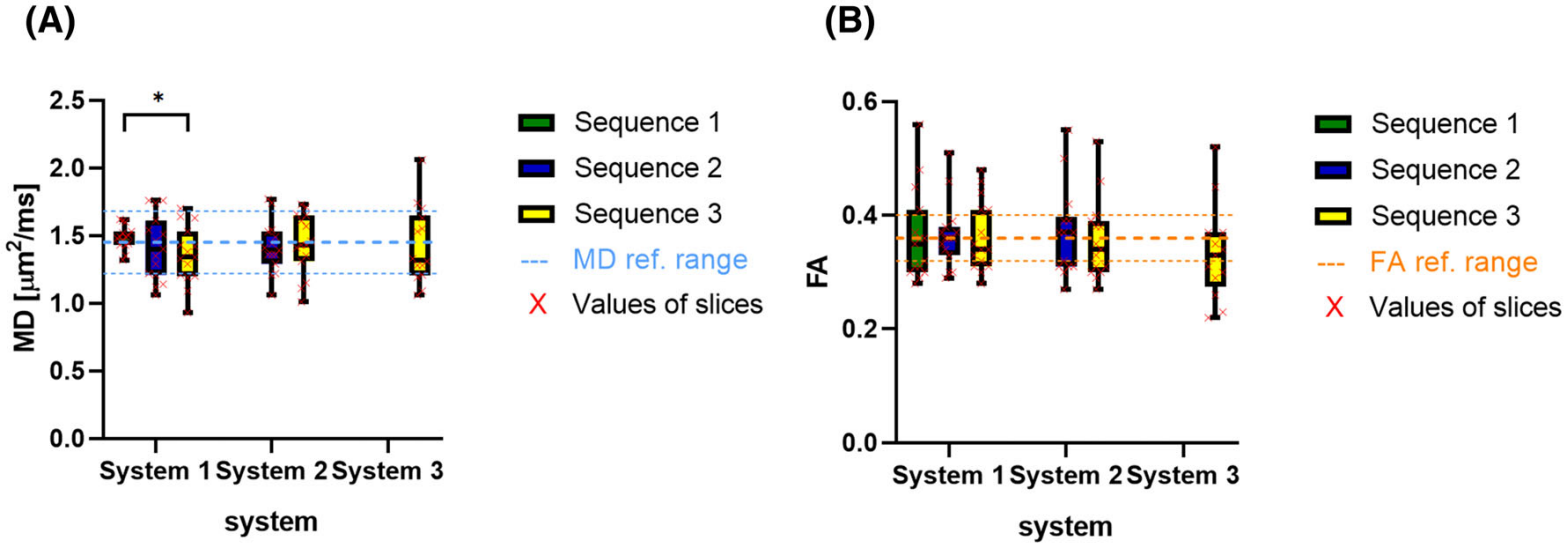
(A) Average left ventricular MD across all volunteers and sequences compared to the mid systolic reference range. (B) Average left ventricular FA across all volunteers and sequences compared to the mid systolic reference range. Both plots indicate median, IQR, and minimum/maximum values.

All FA values are within the literature range of 0.36 ± 0.04 [10], but they are systematically lower than the reference mean, with the lowest mean value being obtained from sequence 3 at system 3 (0.33 ± 0.08). No statistical difference was observed between any of the measurements.

Figure S4 shows MD and FA maps in example slices for all volunteers across all sequence and scanner combinations. Locally elevated MD is observed in combinations using sequence 3 in volunteer 1 and locally elevated FA is observed across different sequence and scanner combinations in volunteer 4.

Figure 6A shows example maps of the orientation of the principal eigenvector of the fitted ellipsoidal diffusion tensor across all sequences and systems in one volunteer. The coordinates relative to the short axis plane are marked by different colors. All maps show broadly similar positioning of *x* and *y*‐dominated areas, with data obtained at system 1 with sequence 1 showing slightly more *z*‐axis components in the endo‐ and epicardial edges of the short axis.

**FIGURE 6:**
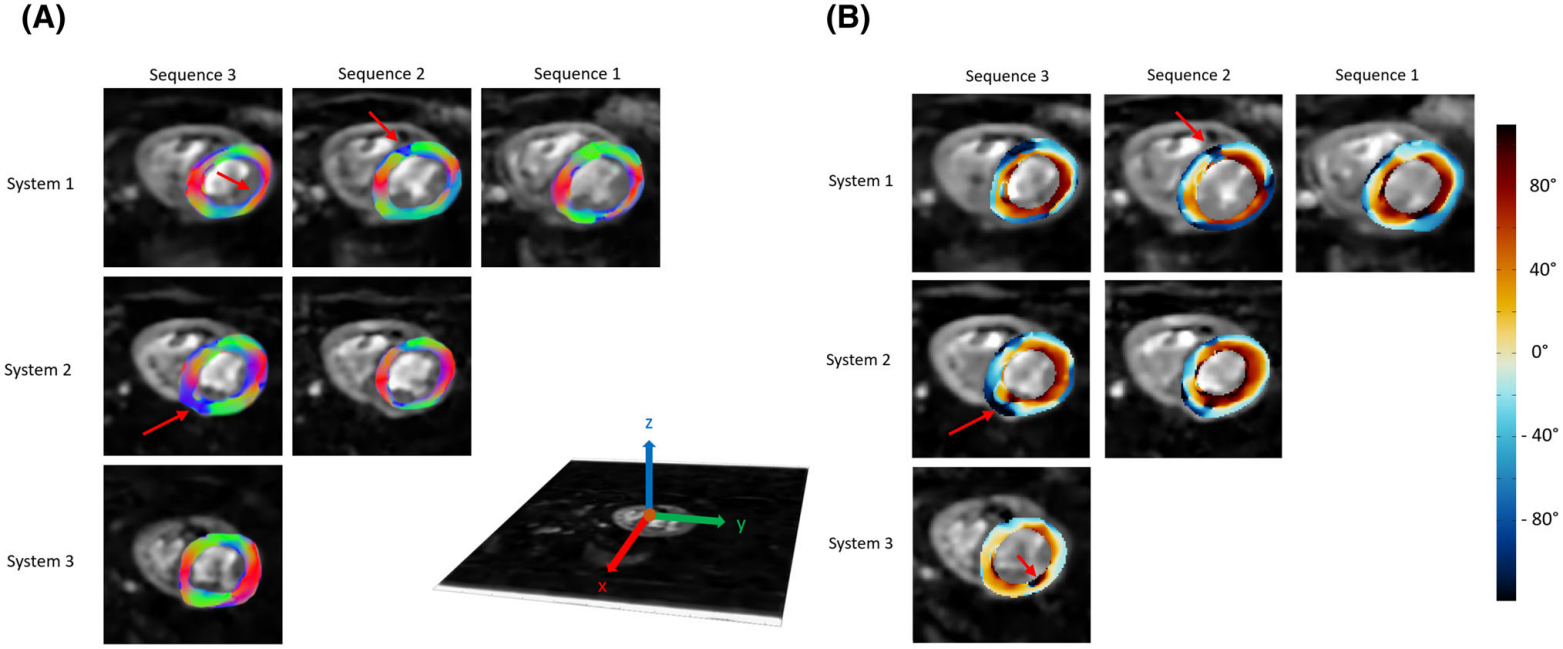
(A) Example primary eigenvector orientation maps of one volunteer. Coordinate orientations in relation to the short axis plane are shown on the bottom right. (B) Example helix angle maps of one volunteer. Observed artifacts are marked with red arrows. Residual fat artifacts often presented themselves as faulty *z*‐directionality or overly negative angles in the epicardial areas. Endocardial presence of *z*‐directionality presumably was caused by blood pool signal contamination.

Figure 6B shows example helix angle maps of the same volunteer. The transmural change of myocyte orientation is apparent in the system 1 images.

Figure S5 shows an example comparison of the original measurement, the retest, and the free‐breathing protocol in one volunteer. Figure S6 shows MD and FA for all three measurement series averaged across all slices in all volunteers, utilizing the same literature reference ranges as in Figure 4. No significant differences were found.

Figure 7 shows maps and histograms of dMD, dFA and dE1 of an example slice obtained at system 1 with sequence 1. Uncertainty values across all scanner and sequence combinations for all volunteers are listed in Table 2. Both the lowest maximum likelihood dMD and lowest median dMD result from sequence 2 applied at system 2, with values of (0.34 ± 0.08) $\mu m^{2}/\mathrm{ms}$ and (0.41 ± 0.06) $\mu m^{2}/\mathrm{ms}$, respectively. dFA is higher relative to the absolute values across every combination, with the lowest median of 0.31 ± 0.06 being obtained at system 1 with sequence 1. A table listing mean dMD values obtained in the phantom across all diffusivity levels can be found in the Table S1. The values range from (0.03 ± 0.01) $\mu m^{2}/\mathrm{ms}$ to (0.06 ± 0.01) $\mu m^{2}/\mathrm{ms}$.

**FIGURE 7:**
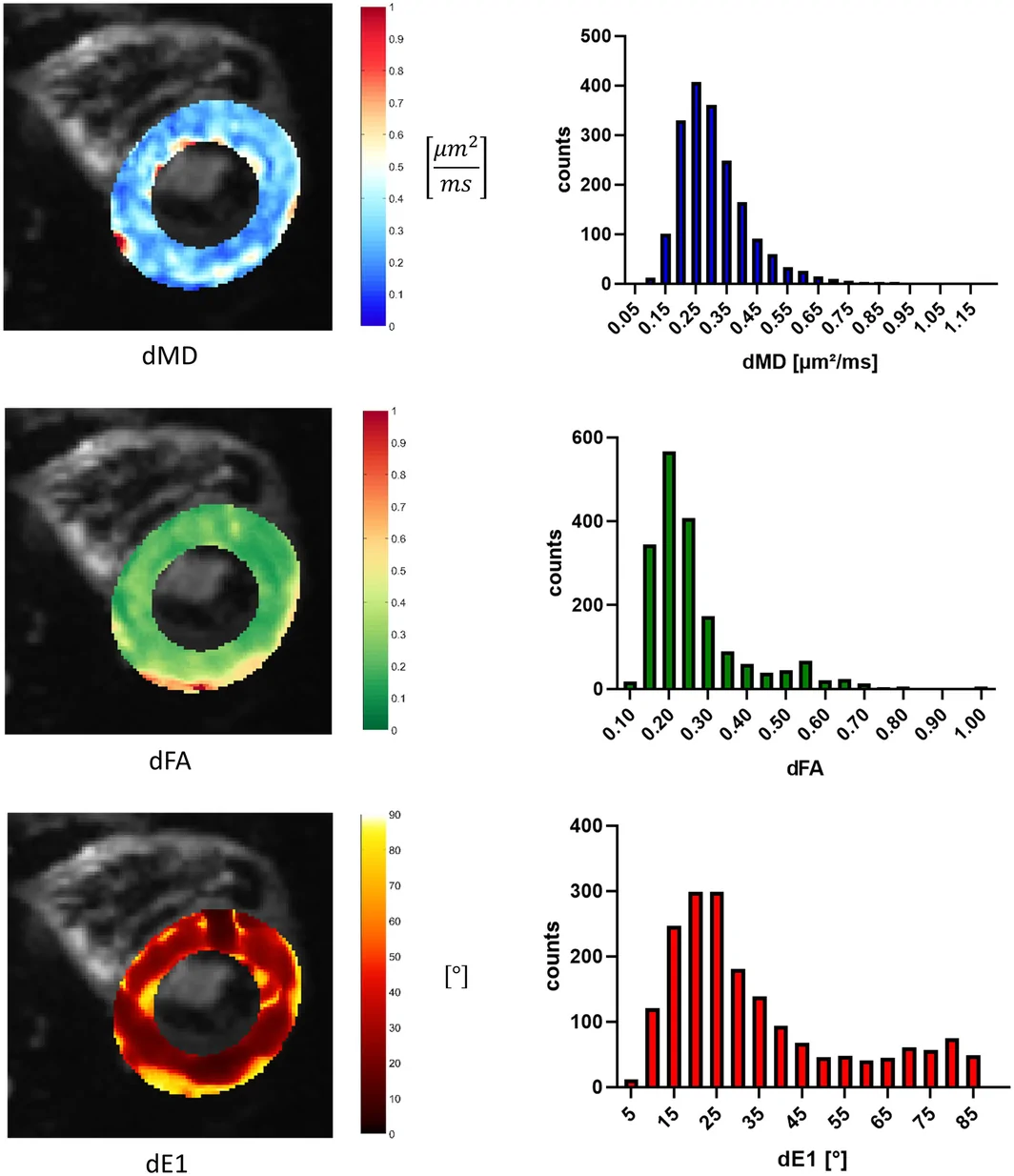
dMD, dFA, and dE1 values obtained from bootstrapping for one example slice. The histograms on the right were obtained from the pixel values in the shown masks and were used to determine the maximum likelihood values.

**TABLE 2:** Maximum likelihood (upper rows) and median (lower rows) uncertainties and their standard deviations of DTI parameters based on 95% CI bootstrapping results.

System/sequence	Sequence 3 Max. likelihood/median uncertainties	Sequence 2 Max. likelihood/median uncertainties	Sequence 1 Max. likelihood/median uncertainties
System 1	(0.37 ± 0.09) $\mu m^{2}/\mathrm{ms}$	(0.44 ± 0.21) $\mu m^{2}/\mathrm{ms}$	(0.44 ± 0.07) $\mu m^{2}/\mathrm{ms}$
	(0.44 ± 0.18) $\mu m^{2}/\mathrm{ms}$	(0.52 ± 0.18) $\mu m^{2}/\mathrm{ms}$	(0.51 ± 0.22) $\mu m^{2}/\mathrm{ms}$
	0.32 ± 0.13	0.32 ± 0.16	0.32 ± 0.03
	0.39 ± 0.10	0.38 ± 0.12	0.32 ± 0.09
	(56.32 ± 32.43) °	(36.21±26.99) °	(36.21±4.67) °
	(50.39 ± 11.40) °	(48.42±14.31) °	(38.67±14.64) °
System 2	(0.39 ± 0.08) $\mu m^{2}/\mathrm{ms}$	(0.34 ± 0.08) $\mu m^{2}/\mathrm{ms}$	
	(0.43 ± 0.09) $\mu m^{2}/\mathrm{ms}$	(0.41 ± 0.06) $\mu m^{2}/\mathrm{ms}$	
	0.31 ± 0.06	0.26 ± 0.06	
	0.34 ± 0.05	0.31 ± 0.06	
	(51.99±28.77) °	(19.60±4.81) °	
	(46.28±12.50) °	(36.98±8.38) °	
System 3	(0.39 ± 0.12) $\mu m^{2}/\mathrm{ms}$		
	(0.44 ± 0.12) $\mu m^{2}/\mathrm{ms}$		
	0.45 ± 0.27		
	0.43 ± 0.12		
	(66.85±26.26) °		
	(57.07±12.10) °		

*Note*: Blue rows show dMD, green rows show dFA and red rows show dE1.

### 3.3 SNR Comparison

Figure 8A shows the grouped tSNR of all volunteers as a comparison between the sequences and systems. Significant differences were observed in intra‐system data of different sequences, with sequence 1 showing the lowest tSNR at system 1: 4.80 ± 0.84 (*p* = 0.001 vs. highest). Sequence 2 shows no significant tSNR differences at system 1: 4.94 ± 0.61 compared to system 2: 5.06 ± 0.55 (*p* = 0.54). Sequence 3 differs significantly in system 1, with a value of 5.22 ± 0.67 at system 1 compared to a value of 4.78 ± 0.78 at system 2 (*p* = 5.03e‐5), or a value of 5.02 ± 0.54 at system 3 (*p* = 0.005).

**FIGURE 8:**
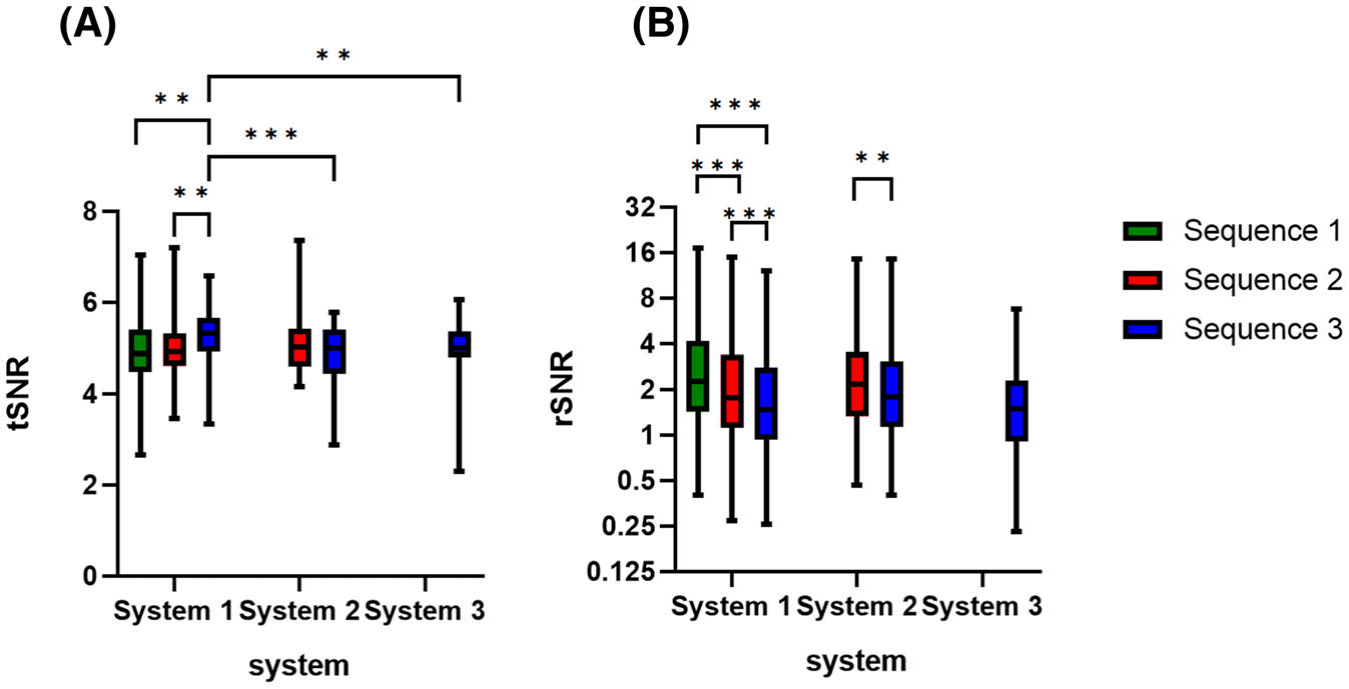
(A) tSNR data of all volunteers. Values were obtained from manually drawn ROIs of the myocardium. (B) rSNR data of all volunteers. Values were obtained from manually drawn ROIs of the myocardium. The *y* axis is plotted as $\log_{2}$ due to the non‐normal distribution of the data. Both plots indicate median, IQR and minimum/maximum values.

Figure 8B shows the averaged rSNR values across all data sets. As expected, sequences with shorter TE show significantly higher rSNR, which is consistent at both system 1 and system 2. Significant differences were observed between sequences 2/3 (2.59 ± 2.21/2.20 ± 1.87) at system 1 (*p* = 3.05e‐5) and 2.82 ± 2.23/2.53 ± 2.22 at system 2 (*p* = 1.0e‐3).

## 4 Discussion

In this work, a systematic comparison of an optimized motion‐compensated cardiac diffusion sequence was performed at three different MRI systems using a vendor‐independent sequence implementation. Images were acquired in the same five volunteers at the three systems using all sequences that could be executed under the given gradient constraints.

DWIs of good quality in terms of SNR and levels of distortion could be acquired with all sequences and scanner configurations. Image quality did vary between averages and slices to a slight degree as is expected from breath‐held cDTI acquisitions but this effect was minor which may be a consequence of the fact that the volunteers were familiar with the principles of MRI acquisitions and were thus avoiding artifact causing movements to a higher degree than expected for clinical patients. Despite the STIR pulse and inner volume excitation, some residual fat signal remained at the chest wall, but it did not overlap with the myocardial areas in most cases due to the chosen phase encoding direction. Due to the inner volume excitation and through plane motion typically present in short axis acquisitions, spin history artifacts are a possible concern. Although the slice gap of 3 mm as well as the inherent spoiling effects of bipolar M2 compensated gradient waveforms may aid in reducing potential artifacts, additional mitigation of spin history or coherence pathway effects caused by out of slice saturation is desirable. Interleaved multislice acquisitions may benefit from the tested flip‐back method by partially compensating for the short repetition time compared to T1. A similar effect could be achieved by triggering the sequence to every other or every third heart beat if acquired in free‐breathing, but at the cost of additional measurement time. Another recently described approach would be the use of oblique, non‐orthogonal slice excitation to avoid the excitation of all slices in the stack on every repetition [46].

The phantom MD results suggest no systematic over‐ or underestimation of MD compared to the references as the measurement values for the two lowest reference MDs were higher than the reference values (difference: (25.6 ± 15.1) *μm* ^2^/s) whereas they were lower for the highest three reference MDs (difference: (114.8 ± 53.9) *μm* ^2^/s).

As expected, reference MD values are temperature dependent; a possible temperature variation of ±1°C was assumed and the resulting uncertainty, represented in the horizontal error bars of Figure 2, was below 5%. The lack of a direct internal temperature measurement does however present a limitation and will be addressed in further studies to provide more accurate reference values.

The calculated in vivo mean DTI values were mostly in line with the literature suggesting the efficacy of the employed post processing pipeline and sufficient quality of the DWI inputs. However, some slices showed very low MD values and high degrees of FA heterogeneity as seen in Figure S4.

The consensus statement [10] was used as a reference for the literature ranges of MD and FA, given that these values depend on the specific sequence implementation. The reference values were also measured with motion compensated spin echo sequences during systole, comparable to this study. Residual fat artifacts were observed that lead to locally elevated FA and decreased MD values. Additional endocardial artifacts were likely caused by blood pool signal contamination. The implementation of a dark blood method may mitigate this effect in future work. Furthermore, parameter uncertainties, in particular dE1 and dFA, were found to be larger compared to prior work [41], which may be related to the lower number of diffusion directions used in this work (9 compared to 12). For FA, the values in noisy areas or areas of potential artifacts were higher than in the myocardium, explaining occasional elevated FA levels as seen in Figure 4B for sequence 3 at system 3 or more broadly in volunteer 4 as seen in Figure S4. Despite this, FA appears to be systematically lower than the literature value, although still within the range of errors. This is likely explained by the differences in observed FA for stimulated echo acquisition mode (STEAM) and SE sequences [10] and possibly by the difference in applied b value. The young average age of the volunteers compared to usual patient cohorts may also influence the results further. The relatively long trigger delay necessitated by the STIR approach may have caused variations in measured cardiac cycle positions between volunteers with differing heart rates, although the effect was assumed to be minor based on the relative stability of the duration of the systole as mentioned prior. The delay was kept constant as increasing it beyond the required STIR inversion time could have moved the acquisition window outside of the systole. A more sophisticated triggering approach such as using a reference point prior to the R wave or using cine imaging to determine the systole [47] could be useful to render timing more flexible in future applications. The differences in orientation between *b*_ref_ and the diffusion directions may also influence the DTI results. The principal eigenvector orientations agree with expected results for a helical structure in terms of forming opposed structures with a 90° offset between each other [10], showing predominantly *x*‐axis orientations in the septal and opposed lateral regions of the left ventricle and *y*‐axis orientations in the posterior and anterior regions in this particular slice orientation. Both the principal eigenvector maps and the helix angle maps conform more closely to expected structures in system 1 acquisitions which can likely be attributed to higher SNR. Helix angle maps show the expected transmural pattern although some artifacts are present. A larger number of diffusion directions [48] as well as a higher resolution [49] would likely improve the maps further. As demonstrated in the recent consensus paper [10], excluding slices with a rate of up to 30% can result in higher quality helix angle maps. In this work no data was excluded for helix angle map estimation in order to avoid potential biasing of the comparisons of different scanner and sequence combinations.

The retest showed no significant differences in MD and FA, indicating appropriate reproducibility. The free‐breathing protocol, including the flip‐back technique, showed no visible motion‐related artifacts, indicating that the proposed sequence together with respiratory binning can be a suitable approach for further studies that may require free‐breathing. More sophisticated methods of free‐breathing motion correction could further improve image quality and increase acquisition efficiency [50].

The feasibility of using higher *b* values was also demonstrated in the flip‐back measurement. This is expected due to reduced perfusion effects that are not accounted for by standard monoexponential diffusion tensor fitting [51]. Additionally, a recent study indicated that higher *b* values can produce more uniform parameter maps [52]. However, higher *b* values are particularly difficult to implement at systems with lower gradient performance due to the longer TE. Therefore, a *b* value of 350 s/mm^2^ was chosen for the main comparison of this study to achieve appropriate SNR at system 3 with only 40 mT/m gradient amplitude.

The mean values of MD and FA derived from the acquisition with the flip‐back technique across all slices showed no significant differences compared to either the retest or the original data. This aligns with the findings of the original publication comparing the flip‐back method to standard inner volume excitation methods [27] and therefore indicates that this may be a valuable technique increasing SNR and reducing artifacts in multislice cDTI with reduced FOV. Both MD and FA obtained from the flip‐back measurements were slightly lower than the values obtained from the original measurements but they are within the IQR of the original flip‐back publication values [27]. The MD values measured with the free‐breathing flip‐back technique show a larger variation compared to the breath‐held measurements without flip‐back, which may be related to the smaller number of effective averages available after retrospective gating, thus yielding lower SNR. This may also explain the observed difference in MD as noise floor effects are known to bias DTI quantities [53], although this effect depends on specific sequence parameters. The more precise matching of trigger delay times to individual subject cardiac cycles in the flip‐back implementation compared to the original acquisitions may also play a part in explaining MD differences, as MD has been reported to change based on cardiac cycle position [54].

The impact of changing the orientation of *b*_ref_ to have equal components in *x*, *y* and *z* compared to only having a *z* component did not result in directly observable differences in the obtained maps. However, spreading the error caused by the difference in orientation between acquisitions of *b* and *b*_ref_ may improve quantitative fidelity in further studies, therefore, the reference direction in *x*‐*y*‐ and *z*‐direction will be set as default in the proposed sequence framework moving forward. The results for dMD from the bootstrapping analysis are slightly elevated compared to literature [41], with the maximum likelihood values being in a range of (0.34 ± 0.08) $\mu m^{2}/\mathrm{ms}$ to (0.52 ± 0.17) $\mu m^{2}/\mathrm{ms}$. Both the lowest maximum likelihood value of (0.34 ± 0.08) $\mu m^{2}/\mathrm{ms}$ and the lowest median value (0.41 ± 0.06) $\mu m^{2}/\mathrm{ms}$ were both obtained at system 2, suggesting a higher degree of temporal stability compared to system 1. The larger values of dE1 indicate larger variation across the averages and slices. The dMD values obtained from the phantom were on the order of (0.04 ± 0.01) $\mu m^{2}/\mathrm{ms}$, representing uncertainties that can be attributed to stochastic and hardware related factors independent of variations present in vivo. This value corresponds to a low single digit percentage error of typical myocardial MD values.

At system 1, rSNR differences of the three sequences follow the theoretically expected decreasing trend with increasing TE. For a human myocardial $T_{2}$ of 54 ms at 3 Tesla [9] and the TEs of the respective sequences, rSNR ratios of $\frac{{\text{rSNR}}_{\mathrm{Seq}1}}{{\text{rSNR}}_{\mathrm{Seq}2}}=1.37$ and $\frac{{\text{rSNR}}_{\mathrm{Seq}2}}{{\text{rSNR}}_{\mathrm{Seq}3}}=1.25$ are expected, whereas the measured ratios are 1.24 and 1.18 respectively. This may be related to the influences on SNR by diffusion weighting and parallel imaging. A further shortening of TEs may be achieved by arbitrary diffusion gradient waveform design to lessen the impact of PNS limitations [55, 56]. Although the obtained tSNR and rSNR values show relatively large standard deviations, significant differences could be observed due to the large number of samples in each data set: multiple slices and diffusion directions were measured in each volunteer, and 150 data points were available for each combination of sequences and MR system. Notably, tSNR was lower at system 1, which is in contrast to the increase in rSNR and also consistent with the dMD findings. This may be caused by greater eddy current artifacts at higher gradient strengths or other system‐specific temporal instabilities, necessitating further investigations of hardware stability. Taking into account the gradient impulse response functions of individual systems may yield more consistent results [57]. The imaging protocol has been shown to be applicable for DTI of early myocardial injury. Longitudinal large animal studies may be performed that combine cardiac DTI with other quantitative metrics such as ^19^F based inflammation imaging [58, 59, 60]. Cardiac DTI could particularly support findings in the context of Interventional MRI of the early myocardial injury [61, 62].

Some limitations were present in the study. The volunteer cohort was small and homogeneous in terms of age and general health. However, acquiring five averages of nine diffusion directions and a reference across three slices for six sequence and system combinations yielded a total of 900 images available for analysis across all five volunteers. Similarly, the bootstrapping analysis was performed for reliable uncertainty quantification based on the available data. Additional data from a broader range of volunteers would be beneficial for future work. Furthermore, myocardial health was assumed based on a questionnaire but not validated through a complete CMR exam. Although three different clinical 3T scanners were examined, they were all built by a single vendor. Comparing scanners across vendors as well as comparing the same scanner type at two different centers could yield further insight into potential sources of systematic deviations of DTI results. No vendor sequence with M2 compensated diffusion gradients suitable for in vivo cDTI comparisons was available. The sequence used in this work utilized parallel imaging which renders the SNR dependent on the coil geometry [43]. Comparing the results to a sequence that does not utilize parallel imaging could serve to better isolate the impact of coil geometry. Comparing the used SE approach to a STEAM based sequence as well as a vendor implementation of cDTI in future work could yield more precise DTI comparisons than presented here.

## 5 Conclusion

The efficacy of the sequence design was demonstrated for acquiring undistorted images of good SNR suitable for further DTI analysis. The vendor‐independent Pulseq code could be useful for research groups with limited experience in cardiac DTI. All three sequence variants could be executed at the systems they were designed for, and differences between the results at the same system followed theoretically expected trends. Significant SNR and MD differences of the same sequence applied to different systems were observed. Knowledge obtained about these differences and the existence of the performance framework used in this work opens the door to potential future ways of solving these discrepancies. This is an important step to ensure the comparability of quantitative cDTI values obtained at different scanners or sites, which is uniquely enabled by the precompiled, open‐source approach to sequence programming.

## Funding

This work was supported by Deutsche Forschungsgemeinschaft, 492563001, 494790146, 566159812, INST 39/1365‐1.

## Conflicts of Interest

Qingping Chen maintains a research collaboration with Siemens Healthineers (Erlangen, Germany). The other authors declare no conflicts of interest.

## Supporting information


**Table S1:** Average dMD values in the phantom across all sequence and scanner combinations. Values of all vials across the range of diffusivities were averaged together.
**Figure S2:** Achievable echo times for different target *b* values across all three used systems.
**Figure S3:** MD results obtained in the diffusion phantom split by system.
**Figure S4:** (A) Mean diffusivities in all volunteers for all sequence and system combinations. (B) Fractional anisotropies in all volunteers for all sequence and system combinations. Observed artifacts are marked with red arrows. Residual fat artifacts often presented themselves as elevated levels of MD or FA in the epicardial areas. Endocardial elevation of MD presumably was caused by blood pool signal contamination.
**Figure S5:** Example of DTI results for the original measurement, the retest and the flip‐back acquisition series in one volunteer 1. Observed artifacts are marked with red arrows. Residual fat artifacts often presented themselves as elevated levels of MD/FA or as *z*‐directionality/negative helix angles in the epicardial areas. Endocardial elevation of MD and HA presumably was caused by blood pool signal contamination. Cine data was not acquired in the original measurement.
**Figure S6:** (A) Mean diffusivity across slices for the original measurement, the retest and the flip‐back acquisition series. No significant differences were determined using a two‐tailed *t*‐test. (B) Fractional anisotropy across slices for the original measurement, the retest and the flip‐back acquisition series. No significant differences were determined using a two‐tailed *t*‐test. Both plots indicate median, IQR and minimum/maximum values.

## Data Availability

The MATLAB code file used to generate the sequences discussed in this work, a list of required dependencies and a reconstruction code example are available on GitHub at https://github.com/Eric-Arbes/cardiac_diffusion.
