# Supplementary material for: Comparative Performance Analysis of an Open Source Motion‐Compensated Cardiac Diffusion Sequence Across Different MRI Systems and Gradient Strengths—A Pilot Study
Source: Magn Reson Med. 2026 Jul 19;96(6):2629–41. doi: 10.1002/mrm.70527 (PMC13619877; doi:10.1002/mrm.70527)
Supplement: Supplementary file 1 [file MRM-96-2629-s001.docx]

| System/Sequence | Sequence 3 | Sequence 2 | Sequence 1 |
| --- | --- | --- | --- |
| System 1 | (0.04$\pm$0.01) ${\mu m^{2}}/{ms}$ | (0.04$\pm$0.01) ${\mu m^{2}}/{ms}$ | (0.04$\pm$0.01) ${\mu m^{2}}/{ms}$ |
| System 2 | (0.03$\pm$0.01) ${\mu m^{2}}/{ms}$ | (0.04$\pm$0.02) ${\mu m^{2}}/{ms}$ |  |
| System 3 | (0.06$\pm$0.02) ${\mu m^{2}}/{ms}$ |  |  |

Supporting Table S1: Average dMD values in the phantom across all sequence and scanner combinations. Values of all vials across the range of diffusivities were averaged together.

Supporting Figure S2: Achievable echo times for different target b values across all three used systems.

Supporting Figure S3: MD results obtained in the diffusion phantom split by system.

Supporting Figure S4: **A** Mean diffusivities in all volunteers for all sequence and system combinations. **B** Fractional anisotropies in all volunteers for all sequence and system combinations. Observed artifacts are marked with red arrows. Residual fat artifacts often presented themselves as elevated levels of MD or FA in the epicardial areas. Endocardial elevation of MD presumably was caused by blood pool signal contamination.

Supporting Figure S5: Example of DTI results for the original measurement, the retest and the flip-back acquisition series in one volunteer 1. Observed artifacts are marked with red arrows. Residual fat artifacts often presented themselves as elevated levels of MD/FA or as z-directionality/negative helix angles in the epicardial areas. Endocardial elevation of MD and HA presumably was caused by blood pool signal contamination. Cine data was not acquired in the original measurement.

Supporting Figure S6: **A** Mean diffusivity across slices for the original measurement, the retest and the flip-back acquisition series. No significant differences were determined using a two-tailed t-test. **B** Fractional anisotropy across slices for the original measurement, the retest and the flip-back acquisition series. No significant differences were determined using a two-tailed t-test. Both plots indicate median, IQR and minimum/maximum values.
